# Predicting LncRNA–Disease Association by a Random Walk With Restart on Multiplex and Heterogeneous Networks

**DOI:** 10.3389/fgene.2021.712170

**Published:** 2021-08-19

**Authors:** Yuhua Yao, Binbin Ji, Yaping Lv, Ling Li, Ju Xiang, Bo Liao, Wei Gao

**Affiliations:** ^1^School of Mathematics and Statistics, Hainan Normal University, Haikou, China; ^2^Key Laboratory of Data Science and Intelligence Education, Ministry of Education, Hainan Normal University, Haikou, China; ^3^Key Laboratory of Computational Science and Application of Hainan Province, Hainan Normal University, Haikou, China; ^4^Geneis Beijing Co., Ltd., Beijing, China; ^5^Basic Courses Department, Zhejiang Shuren University, Hangzhou, China; ^6^School of Computer Science and Engineering, Central South University, Changsha, China; ^7^Department of Basic Medical Sciences, Changsha Medical University, Changsha, China; ^8^Department of Computer Science, Changsha Medical University, Changsha, China; ^9^Departments of Internal Medicine-Oncology, Fujian Cancer Hospital & Fujian Medical University Cancer Hospital, Fuzhou, China

**Keywords:** lncRNA, disease, association, networks, random walk, predict

## Abstract

Studies have found that long non-coding RNAs (lncRNAs) play important roles in many human biological processes, and it is critical to explore potential lncRNA–disease associations, especially cancer-associated lncRNAs. However, traditional biological experiments are costly and time-consuming, so it is of great significance to develop effective computational models. We developed a random walk algorithm with restart on multiplex and heterogeneous networks of lncRNAs and diseases to predict lncRNA–disease associations (MHRWRLDA). First, multiple disease similarity networks are constructed by using different approaches to calculate similarity scores between diseases, and multiple lncRNA similarity networks are also constructed by using different approaches to calculate similarity scores between lncRNAs. Then, a multiplex and heterogeneous network was constructed by integrating multiple disease similarity networks and multiple lncRNA similarity networks with the lncRNA–disease associations, and a random walk with restart on the multiplex and heterogeneous network was performed to predict lncRNA–disease associations. The results of Leave-One-Out cross-validation (LOOCV) showed that the value of Area under the curve (AUC) was 0.68736, which was improved compared with the classical algorithm in recent years. Finally, we confirmed a few novel predicted lncRNAs associated with specific diseases like colon cancer by literature mining. In summary, MHRWRLDA contributes to predict lncRNA–disease associations.

## Introduction

Numerous studies have indicated that protein-coding genes accounted for less than 2% of the human genome (Crick et al., 1961; Yanofsky, 2007). There are many non-translatable RNAs called non-coding RNAs (ncRNAs), which have been considered as transcriptional noise for a long time (Zhang et al., 2017; Xu et al., 2020). Long non-coding RNAs (lncRNAs) whose length are greater than 200 nucleotides are a class of important ncRNAs (Mercer et al., 2009). There are increasing evidence that lncRNAs play key roles in many important biological processes and diseases (Akerman et al., 2017; Wang et al., 2019; Peng et al., 2020). For example, HOTAIR was considered as a potential biomarker for liver cancer (Yang et al., 2011; Li et al., 2019), lung cancer (Li G. et al., 2014a), and colorectal cancer (Kogo et al., 2011; Maass et al., 2014), and UCA1 was a potential biomarker for bladder cancer diagnosis (Zhang et al., 2012). Li J. et al. (2014b) summarized the important role of lncRNA such as MALAT1, HOTAIR, and other specific lncRNAs for hepatocellular carcinoma. LncRNAs associated with tumor immune invasion in non-small cell lung cancer (NSCLC) have important value in improving clinical efficacy and immunotherapy, compared with normal controls, and the expression of gabpb1-it1 was significantly downregulated in NSCLC. In addition, overexpression of gabpb1-it1 in cancer samples is associated with increased survival in NSCLC patients (Sun et al., 2020). Inferring the association between lncRNA and diseases can better study human diseases and help the diagnosis and treatment of diseases, and accelerate the identification of potential drug response predictors (Liu et al., 2016, 2020). Therefore, the exploration of lncRNA–disease association has attracted more and more attention from biologists. The establishment of an effective computational model to predict the association between lncRNAs and diseases can save time and money spent in biological experiments (Yao et al., 2019; Yan et al., 2020).

At present, many machine learning methods have been proposed to predict the lncRNA–disease association, for example, Laplacian regulated least square method (LRLSLDA; Chen and Yan, 2013), propagation algorithm (Yang et al., 2014), a method based on Bayesian classifier (Zhao et al., 2015), and a method based on induction matrix (Lu C. et al., 2018a). However, these machine learning methods need negative samples, which are difficult to obtain. In order to solve this problem, network-based methods emerge as the times require. With the increasing importance of revealing the molecular basis of human diseases, network-based methods have been widely used in exploring disease-related genes (Yan et al., 2015; Hu et al., 2018; Lu M. et al., 2018b; Yang et al., 2020). For example, Xiang et al. (2021) proposed a multibiological network (NIDM) network pulse dynamics framework and a fast network embedding (Xiang et al., 2020) to predict disease-related genes. Network-based algorithms have also been widely studied in predicting lncRNA–disease association. Bellucci et al. (2011) combined the expression similarity of lncRNA with the Gaussian nuclear interaction spectrum similarity of lncRNA, and proposed a potential protein determination method based on sequence information to predict the function of lncRNA. In the study of Xiao et al. (2015), the function of lncRNA was predicted by constructing the regulatory network between lncRNA and protein coding genes. In the BPLLDA study, the authors estimated the potential relationship between disease and lncRNAs by connecting the length of the disease and lncRNA pathway (Xiao et al., 2018). KATZLDA was a computing method to predict lncRNA–disease association based on the similarity between heterogeneous network nodes (Chen, 2015a). The random walk model is also widely used in the field of data mining and Internet, and many researches use this method to predict potential association (Xing et al., 2012; Yang et al., 2016, 2017; Gu et al., 2017). Zhou et al. (2015) proposed a new method by integrating the related lncRNA–lncRNA network, disease–disease similarity network, and the heterogeneous lncRNA–disease association network, and then realized random walk on the heterogeneous network. Sun et al. (2014) proposed a method for constructing lncRNA–lncRNA functional similarity network and then developed a calculation method based on global network (RWRlncD). Recently, Lei and Bian (2020) used random walk to weight the structural features of circRNA–disease pairs and combined it with k-nearest neighbor algorithm to get the prediction score of each circRNA–disease pair. Although these methods have been proposed to predict lncRNA–disease association successfully, it is still a challenge to make full use of multi-source biological data.

In this study, a random walk algorithm with restart on multiplex and heterogeneous networks was developed. The downloaded known lncRNA–disease association data were used to calculate lncRNA functional similarity, lncRNA Gaussian interaction kernel similarity, disease semantic similarity, and disease Gaussian interaction kernel similarity, respectively. Then, these similarity networks and lncRNA–disease association network were constructed into multiplex and heterogeneous networks. A random walk with restart was carried out on the multiplex and heterogeneous networks, and the potential lncRNA–disease association was predicted using the final stable probability.

## Materials and Methods

### LncRNA–Disease Association

LncRNADisease (Chen, 2015b), Lnc2Cancer (Ning et al., 2016), MNDR (Wang et al., 2013), and other databases stored the known lncRNA–disease association data, which have been of great help in predicting novel association. In this study, 285 lncRNA–disease association was downloaded from lncRNADisease database, including 117 lncRNAs and 159 diseases. We used *LD* to represent the lncRNA–disease association adjacency matrix. If lncRNA(*i*) is related to disease(*j*), then *LD*(*i*, *j*) = 1; otherwise, *LD*(*i*, *j*) = 0, that is:

(1) $$LD\left(i,j\right)=\left\{ \begin{array}{ccc} 1, & iflncRNA\left(i\right)isassociatedwithdisease\left(j\right) & \\ 0\; & otherwise & \end{array}\right.$$

### Disease Similarity

#### Disease Semantic Similarity

Directed acyclic graphs (*DAGs*) were used to calculate disease–disease similarity, for disease *d_k_*, let *DAG*(*d*_*k*_, *T*(*d*_*k*_), *E*(*d*_*k*_)) be its directed acyclic graph, where *T*(*d*_*k*_) are ancestor nodes of *d_k_*, and *E*(*d*_*k*_) represents the corresponding set of edges from parent node to child nodes. Semantic similarity of diseases was calculated by *R* package called DOSim (Li et al., 2011); for any disease *k* in *DAG*(*d*_*k*_, *T*(*d*_*k*_), *E*(*d*_*k*_)), the semantic contribution of *k*to *d_k_* was defined as:

(2) $$D_{d_{k}}\left(k\right)=\left\{ \begin{array}{ccc} 1, & ifk=d_{k} & \\ max\left\{0.5*D_{d_{k}}\left(k^{\prime }\right)|k^{\prime }\in childrenofk\right\}, & ifk\neq d_{k} & \end{array}\right.$$

The above formula indicates that the contribution of the disease to its semantic value is 1. Semantic contribution decreased with the increase of the distance between disease *k* and other diseases. Then, the semantic similarity between *d_i_* and *d_j_* was defined as:

(3) $$DSS\left(d_{i},d_{j}\right)=\frac{\sum_{k\in T_{d_{i}}\cap T_{d_{j}}}\left(D_{d_{i}}\left(k\right)+D_{d_{j}}\left(k\right)\right)}{\sum_{k\in T_{d_{i}}}D_{d_{i}}\left(k\right)+\sum_{k\in T_{d_{j}}}D_{d_{j}}\left(k\right)}$$

#### Gaussian Interaction Profile Kernel Similarity for Diseases

In order to obtain the similarity information between diseases, the Gaussian Interaction Profile kernel similarity between disease was constructed based on the lncRNA–disease association network. First, the Interaction Profile (IP) of each disease represents a binary code in the known lncRNA–disease association network. For example, for given disease *d_i_*, its *IP*(*d*_*i*_) represents the *i*th column of *LD*. Next, the Gaussian Interaction Profile kernel similarity between *d_i_* and *d_j_* was calculated as:

(4) $$DS_{GIP}\left(d_{i},d_{j}\right)=exp(-\gamma_{d}||IP\left(d_{i}\right)-IP\left(d_{j}\right)|{|}^{2}$$

Where γ_*d*_ represents the bandwidth that controls the Gaussian Interaction Profile kernel similarity, $\gamma_{d}=\frac{\gamma_{d}^{\prime }}{(\frac{1}{nd}}\sum_{i=1}^{nd}||IP\left(d_{i}\right)|{|}^{2})$; in this study, according to van Laarhoven et al. (2011), we set $\gamma_{d}^{\prime }=1$, and *nd* represents the number of diseases.

### LncRNA Similarity

#### LncRNA Functional Similarity

Studies have shown that similar lncRNAs are usually associated with similar diseases. Therefore, lncRNA functional similarity can be roughly estimated by their similarity in related diseases (Sun et al., 2014). For any two lncRNAs *l_i_* and *l_j_*, *D*_*i*_ = {*d_i_k__*|1 ≤ *k* ≤ *m*} and *D*_*j*_ = {*d_j_l__*|1 ≤ *l* ≤ *n*} were disease sets associated with *l_i_* and *l_j_*, respectively. The semantic similarity between disease *d* and disease set *D* was firstly defined as:

(5) $$SS\left(d,D\right)=\underset{d_{l}\in D}{max}DSS\left(d,d_{l}\right)$$

Then, the functional similarity between *l_i_* and *l_j_* was defined as:

(6) $$NFS\left(l_{i},l_{j}\right)=\frac{\sum_{i=1}^{m}SS\left(d_{i_{a}},D_{j}\right)+\sum_{j=1}^{n}SS\left(d_{j_{b}},D_{i}\right)}{m+n}$$

#### Gaussian Interaction Profile Kernel Similarity for LncRNAs

Similar to the disease Gaussian interaction profile kernel similarity. The formula for calculating the Gaussian interaction profile kernel similarity between *l_i_* and *l_j_* was:

(7) $$LS_{GIP}\left(l_{i},l_{j}\right)=exp\left(-\gamma_{l}{||IP\left(l_{i}\right)-IP\left(l_{j}\right)||}^{2}\right)$$

Where γ_*l*_ represents the bandwidth that controls the property similarity of Gaussian interaction kernel, $\gamma_{l}=\frac{\gamma_{l}^{\prime }}{(\frac{1}{nl}}\sum_{i=1}^{nl}||IP\left(l_{i}\right)|{|}^{2})$; in this study, $\gamma_{l}^{\prime }=1$, *nl* represents the number of lncRNAs, *IP*(*l*_*i*_) and *IP*(*l*_*j*_) represent the *i*th and *j*th row of the *LD*, respectively.

### A Random Walk With Restart on Multiplex and Heterogeneous Networks

**FIGURE 1:**
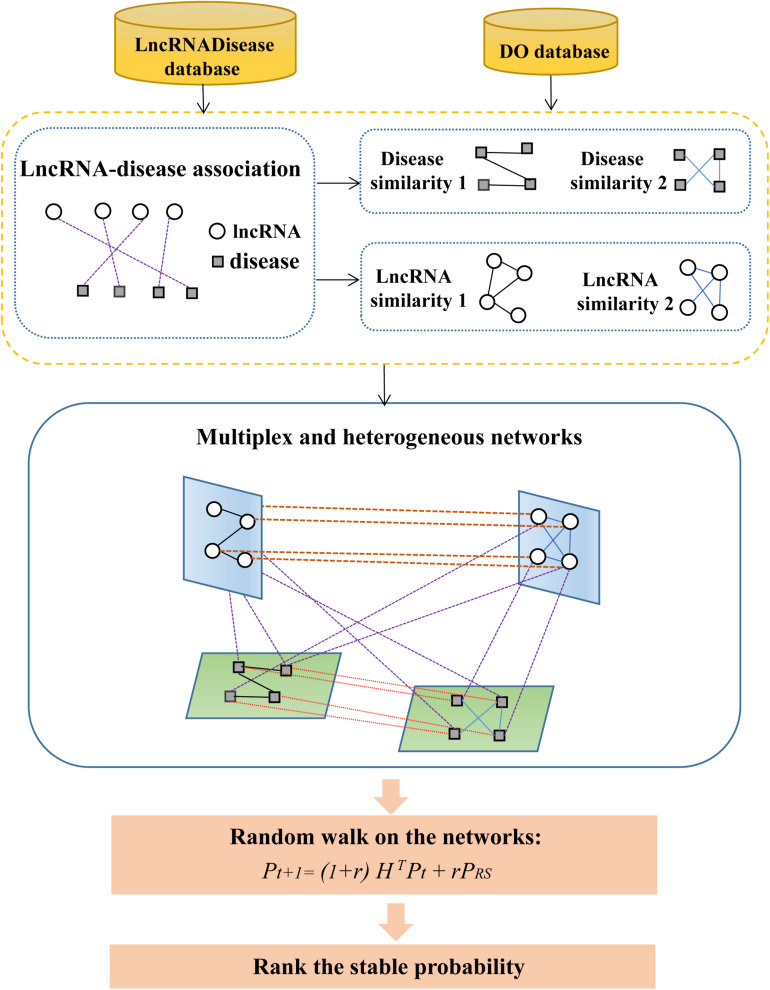
The framework of MHRWRLDA.

An overview of MHRWRLDA is shown in Figure 1. Specifically, we first downloaded the data of known lncRNA–disease association from the LncRNADisease database and got diseased’ DO ID from the DO database to calculate disease similarity. After compute disease similarity and lncRNA similarity, a multiplex and heterogeneous network was set up based on these similarity networks and known lncRNA–disease association network. Finally, a random walk algorithm with restart was implemented on networks, and the final stability probability was used to conduct the predictions.

#### Multiplex and Heterogeneous Network

Based on disease semantic similarity network, disease Gaussian similarity network, lncRNA similarity network, and lncRNA Gaussian similarity network, we constructed a multiplex and heterogeneous network by using lncRNA–disease association. In these networks, the set of lncRNA nodes was defined as: $R_{M}=\left\{v_{i}^{\alpha },i=1,2,\cdots ,n;\alpha =1,2,\cdots ,L\right\}$, where $v_{i}^{\alpha }$ represents the *i*th node on the α layer. The set of disease nodes was defined as: $D_{M}=\left\{v_{j}^{\beta },j=1,2,\cdots ,m;\beta =1,2,\cdots ,K\right\}$, where $v_{i}^{\beta }$ represents the *j*th node on the β layer. The adjacency matrix on each layer is:

(8) $$\begin{array}{c} A^{\left[\alpha \right]}=A^{\left[\alpha \right]}\left(i,j\right)\\ \;=\left\{ \begin{array}{cc} 1,ifthei^{th}nodeisassociatedwith & \\ thejthnodeonlayer\alpha & \\ 0,\;otherwise & \end{array}\right. \end{array}$$

A particle can either travel from the previous node $v_{i}^{\alpha }$ term to any neighbor node on the same layer, or it can also jump to the same node on a different layer. The matrix *A* contains different types of jumps that the particle can follow at each step:

(9) $$A=\left(\begin{array}{cc} \begin{array}{cc} \left(1-\delta \right)A^{\left[1\right]} & \frac{\delta }{\left(L-1\right)}I\\ \frac{\delta }{\left(L-1\right)}I & \left(1-\delta \right)A^{\left[2\right]} \end{array} & \begin{array}{cc} \cdots & \frac{\delta }{\left(L-1\right)}I\\ \cdots & \frac{\delta }{\left(L-1\right)}A \end{array}\\ \begin{array}{cc} \vdots & \vdots \\ \frac{\delta }{\left(L-1\right)}I & \frac{\delta }{\left(L-1\right)}I \end{array} & \begin{array}{cc} \ddots & \vdots \\ \cdots & \left(1-\delta \right)A^{\left[L\right]} \end{array} \end{array}\right)$$

Where *I* is the *n* × *n* identity matrix, the diagonal element of *A* represents the particle walking on same layer, the off-diagonal element represents the particle jumping between different layers, and the parameter δ ∈ (0, 1) represents the probability of the particle walking on the same layer or jumping between different layers. If δ = 0, the particles will always walk on the same layer.

*A*_*RM(nL × nL)*_, *A*_*DM(mK × mK)*_ is the matrix of lncRNA similarity and disease similarity on multiplex and heterogeneous networks, respectively. *n*, *L*, *m*, and *K* are the number of lncRNAs, lncRNA similarity networks, diseases, and disease similarity networks, respectively, the adjacency matrix is: *B*_*MH*_ = (*B*_*n* × *m*_, *B*_*n* × *m*_, ⋯, *B*_*n* × *m*_)^*T*^.

The dimension of *B*_*MH*_ is *nL* × *mK*, which is equivalent to replicating the adjacency matrix *B*_*n* × *m*_ *L*^∗^*K* times, where *B* = *LD*. Then, the adjacency matrix of the whole multiplex and heterogeneous networks is: $A=\left[\begin{array}{cc} A_{RM} & B_{MH}\\ B_{MH}^{T} & A_{DM} \end{array}\right]$.

#### Random Walk With Restart on Multiplex and Heterogeneous Networks

A random walk with a restart means that a particle starts at a node and it is faced with two choices at each walk: move to a randomly selected neighbor node, or jump back to the start node. Considering the time is discrete, *t* ∈ *ℕ*, the particle is at node *v_t_* at the *t*th step. Then, it walks from *v_t_* to *v*_*t*1_. We defined a restart probability γ ∈ (0, 1), and the random walk with restart can be defined as:

(10) $$P_{t+1}=\left(1-\gamma \right)H^{T}P_{t}+\gamma P_{RS}$$

Where the vectors *P*_*t*1_ and *P_t_* represent the probability distribution of *v_t_* and *v*_*t*1_, respectively. *P*_*RS*_ is the initial probability distribution and $P_{RS}=\left[\begin{array}{c} (1-\eta )R_{0}\\ \eta D_{0} \end{array}\right]$; the importance of each network is adjusted by adjusting *P*_*RS*_, where *R*_0_ and *D*_0_ represent the initial probability distribution of lncRNA similarity network and disease similarity network, respectively, and the dimensions of the vectors *P*_*t*+1_, *P_t_*, and *P*_*RS*_ are *nL* × *mK*. The parameter η ∈ (0, 1) controls the probability of each network restarting; if η < 0.5, the particle is more likely to be restarted in lncRNA similarity networks. $H=\left[\begin{array}{cc} H_{RR} & H_{RD}\\ H_{DR} & H_{DD} \end{array}\right]$ represents the transition probability matrix of multiplex and heterogeneous networks, where *H*_*RR*_ and *H*_*DD*_ represent the transition probability of nodes upstream in the same layer, *H*_*RD*_ and *H*_*DR*_ represent the transition probability of node jump between different layers. For a given node, if dichotomous correlation exists, the particle can jump between layers or stay in the current layer with probability λ ∈ (0, 1), and the closer it is to 1, the higher the probability of jumping between different networks.

We suppose a particle was located at the node *r*_*i*_ ∈ *R*. In the next step, the particle can walk to the node *r*_*j*_ ∈ *R*. The transfer probability is:

(11) $$H_{RR}=\left\{ \begin{array}{cc} \frac{A_{R}\left(i,j\right)}{\sum_{k=1}^{n}A_{R}\left(i,k\right),} & \text{if}\sum_{k=1}^{m}B\left(i,k\right)=0\\ \left(1-\lambda \right)\frac{A_{R}\left(i,j\right)}{\sum_{k=1}^{n}A_{R}\left(i,k\right),} & \text{otherwise} \end{array}\right.$$

It can also jump to the node *d*_*b*_ ∈ *D* through binary correlation, and the transfer probability is:

(12) $$H_{RD}=\left\{ \begin{array}{cc} \frac{\lambda B\left(i,b\right)}{\sum_{k=1}^{m}B\left(i,k\right),} & \text{if}\sum_{k=1}^{m}B\left(i,k\right)\neq 0\\ 0 & \text{otherwise} \end{array}\right.$$

Similarly, if the particle was located at the node *d*_*a*_ ∈ *D*, then the transfer probability of the particle walking to the node *d*_*b*_ ∈ *D* is:

(13) $$H_{DD}=\left\{ \begin{array}{cc} \frac{A_{D}\left(a,b\right)}{\sum_{k=1}^{m}A_{D}\left(a,k\right),} & \text{if}\sum_{k=1}^{n}B\left(k,b\right)=0\\ \left(1-\lambda \right)\frac{A_{D}\left(a,b\right)}{\sum_{k=1}^{m}A_{D}\left(a,k\right),} & \text{otherwise} \end{array}\right.$$

If the particle jumps to the node *r*_*j*_ ∈ *R* through binary correlation, then the transfer probability is:

(14) $$H_{DR}=\left\{ \begin{array}{cc} \frac{\lambda B\left(j,a\right)}{\sum_{k=1}^{n}B\left(k,a\right),} & \text{if}\sum_{k=1}^{n}B\left(k,a\right)\neq 0\\ 0, & \text{otherwise} \end{array}\right.$$

When predicting lncRNAs that are potentially associated with the given disease *d_i_*, the node *d_i_* will be used as the seed node in disease similarity networks. The initial probability *D*_0_ is 1 for the given node *d_i_* and 0 for the remaining nodes. If there are known associations among lncRNAs *r*_1_, *r*_2_ ⋯ and disease *d_i_*, then the nodes *r*_1_, *r*_2_ ⋯ are the seed nodes in lncRNA similarity networks. The initial probability *R*_0_ was assigned to seed node *r*_1_, *r*_2_ ⋯, with a probability of 1, and the remaining nodes were 0. *P_t_* converges after some iteration, that is, *P*_*t*_ − *P*_*t* + 1_ < 10^−10^, and we denoted the stable probability as: $P_{\infty }=\left[\begin{array}{c} (1-\eta )R_{\infty }\\ \eta D_{\infty } \end{array}\right]$.

Based on the stabilized *R*_∞_, those seed nodes *r*_1_, *r*_2_ ⋯ were removed, and the remaining lncRNAs were ranked. The higher the ranked lncRNA, the more likely it was to be associated with the given disease *d_i_*. Similarly, a lncRNA can also be designated to predict diseases related to it.

## Results

### Indicators of Performance Evaluation

For a binary classification problem, the confusion matrix is shown in Table 1. Precision, specificity, and sensitivity are evaluation indicators of classification models. They are calculated as:

$$FPR=1-\text{specificity}=\frac{FP}{TN+FP}$$

$$TPR=\text{sensitivity}=\frac{TP}{TP+FN}$$

**TABLE 1:** Confusion matrix definitions.

True prediction	Positive	Negative
Positive	True positive (TP)	False positive (FP)
Negative	False negative (FN)	True negative (TN)

To evaluate the performance of MHRWRLDA, the receiver operating characteristic (ROC) curve was drawn by calculating TPR and FPR according to different thresholds. Area under the curve (AUC) is the area under the ROC curve, and this area is less than 1. Since the ROC curve cannot directly indicate which classifier has better effect in many cases, as a value, the larger the AUC is, the better the classifier has an effect.

### Performance of MHRWRLDA

In order to evaluate the performance of MHRWRLDA for predicting lncRNA–disease association, we applied the known lncRNA–disease association data to MHRWRLDA, and used Leave-One-Out cross-validation (LOOCV) to verify. For global LOOCV, the scores of all test samples are compared with those of all candidate samples. For local LOOCV, each known lncRNA related to a particular disease is selected as the test sample, and other related lncRNAs are selected as the training samples; the scores of test samples are only compared with those of candidate samples. In this study, there are a total of three parameters, namely, γ, λ, and η, and their range is (0, 1), where γ is the restart probability; λ is the jump probability, reflecting the probability of particles jumping between different networks; and η regulated the probability of each network restarting, When η = γ = 0.9 and λ = 0.9, the prediction effect is the best; at this point, AUC = 0.68736.

**FIGURE 2:**
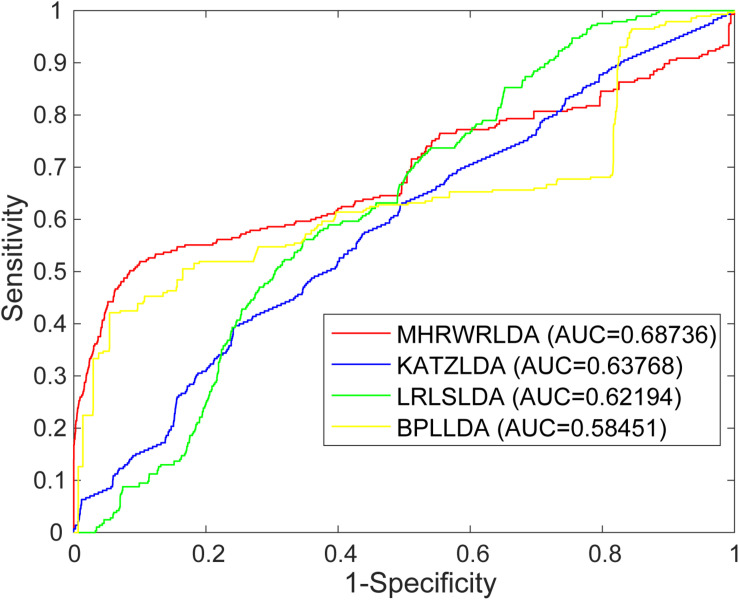
The ROC curves of MHRWRLDA, KATZLDA, BPLLDA, and LRLSLDA based on global LOOCV.

**FIGURE 3:**
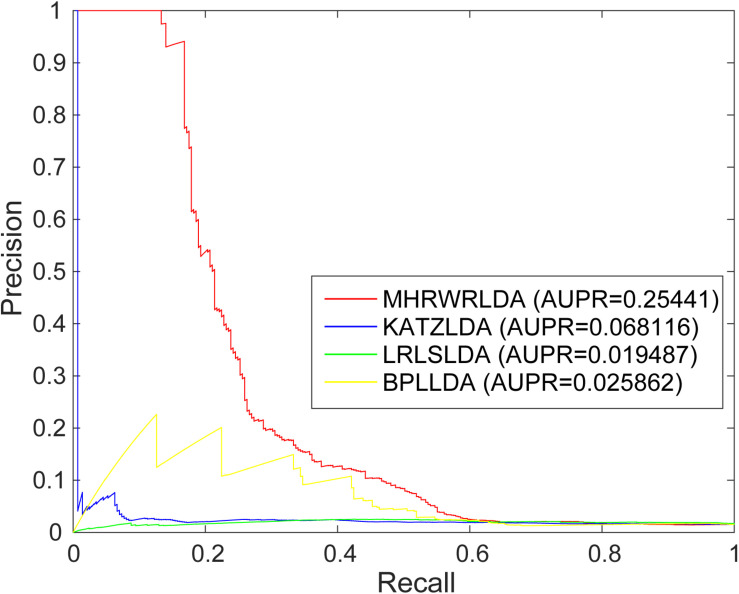
The PR curves of MHRWRLDA, KATZLDA, BPLLDA, and LRLSLDA based on global LOOCV.

The AUC based on global LOOCV of the KATZLDA (Chen, 2015a), BPLLDA (Xiao et al., 2018), and LRLSLDA (Chen and Yan, 2013) were 0.63768, 0.5845, and 0.6219, respectively. The ROC curves of MHRWRLDA, KATZLDA, BPLLDA, and LRLSLDA based on global LOOCV are shown in Figure 2, the PR curves based on global LOOCV are shown in Figure 3, and the AUPR values are shown in their legends. Their ROC curves and PR curves based on local LOOCV are shown in Supplementary Figures 1, 2. The results showed that MHRWRLDA performed better than other classical algorithms in predicting lncRNA–disease association.

### Case Study

To further explore the performance of MHRWRLDA in predicting lncRNA–disease association, we selected colon cancer, hepatocellular carcinoma, and breast cancer for the case study. During the experiment, all known associations were considered as the train set, and unknown associations were regarded as the test set. According to LOOCV results, we sorted lncRNAs and selected the top 10 lncRNAs for further verification based on the LncRNADisease database and several recently published studies.

Colon cancer is a malignant tumor, causing nearly 700,000 deaths each year, and has a high incidence rate record in developed countries. We applied MHRWRLDA to colon cancer experiments to predict the top 10 lncRNAs related to colon cancer (Table 2). Seven of the top 10 lncRNAs have been confirmed in databases or other literature. Previous studies have found that the third ranked CDKN2B-AS1 up-regulates HCT116, thereby causing cell proliferation (Chiyomaru et al., 2013). In addition, studies have shown that removal of PVT1 (ranked 5) from MCY-driven colon cancer strain HCT116 can reduce carcinogenicity (Tseng et al., 2014).

**TABLE 2:** The predicted top 10 lncRNAs for colon cancer.

Disease	Rank	LncRNA	Evidence
Colon cancer	1	H19	Confirmed
	2	MEG3	Confirmed
	3	CDKN2B-AS1	Confirmed
	4	MALAT1	Confirmed
	5	PVT1	Confirmed
	6	BCYRN1	Unknown
	7	IGF2-AS	Confirmed
	8	Anti-NOS2A	Unknown
	9	WT1-AS	Unknown
	10	UCA1	Confirmed

Hepatocellular carcinoma is one of the most common cancers in the world. Studies have shown that hepatocellular carcinoma is the main component of primary liver cancer. We listed the top 10 lncRNAs related to hepatocellular carcinoma predicted by experiments in Table 3. Of the top 10, 9 were all verified in known databases. The overexpression of CDKN2B-AS1, which ranked 8, can inhibit the proliferation and invasion of liver cancer cells (Hua et al., 2015), thereby promoting the apoptosis of liver cancer cells and preventing the occurrence of hepatocellular carcinoma. Ding et al. identified PVT1 (ranked 9) as a novel biomarker for predicting tumor recurrence in patients with hepatocellular carcinoma (Ding et al., 2015).

**TABLE 3:** The predicted top 10 lncRNAs for hepatocellular carcinoma.

Disease	Rank	LncRNA	Evidence
Hepatocellular carcinoma	1	H19	Confirmed
	2	MEG3	Confirmed
	3	MALAT1	Confirmed
	4	AIR	Confirmed
	5	HULC	Confirmed
	6	HOTAIR	Confirmed
	7	IGF2-AS	Confirmed
	8	CDKN2B-AS1	Confirmed
	9	PVT1	Confirmed
	10	BCYRN1	Unknown

Breast cancer accounts for 22% of all cancers in women and is the second leading cause of cancer death in women (Donahue and Genetos, 2013; Karagoz et al., 2015). Traditionally, breast cancer has been diagnosed on the basis of histopathological features such as tumor size, grade, and lymph node status. The prediction of breast cancer-related lncRNAs may help diagnose and treat breast cancer (Meng et al., 2014). In order to diagnose and treat breast cancer better, it is necessary to predict lncRNAs associated with breast cancer and identify lncRNA biomarkers (Xu et al., 2015). We implemented MHRWRLDA on breast cancer to predict potentially relevant lncRNAs, and listed the top 10 lncRNAs related to breast cancer in Table 4. The downregulation of the top ranked first H19 significantly reduced breast cancer clonal formation and anchored independent growth (Barsytelovejoy et al., 2006). In addition, the incidence of breast cancer is also affected by PVT1 overexpression due to genomic abnormalities (Guan et al., 2007).

**TABLE 4:** The predicted top 10 lncRNAs for breast cancer.

Disease	Rank	LncRNA	Evidence
Breast cancer	1	H19	Confirmed
	2	CDKN2B-AS1	Confirmed
	3	PVT1	Confirmed
	4	MEG3	Confirmed
	5	BCYRN1	Confirmed
	6	SRA1	Confirmed
	7	XIST	Confirmed
	8	GAS5	Confirmed
	9	HOTAIR	Confirmed
	10	DSCAM-AS1	Confirmed

**FIGURE 4:**
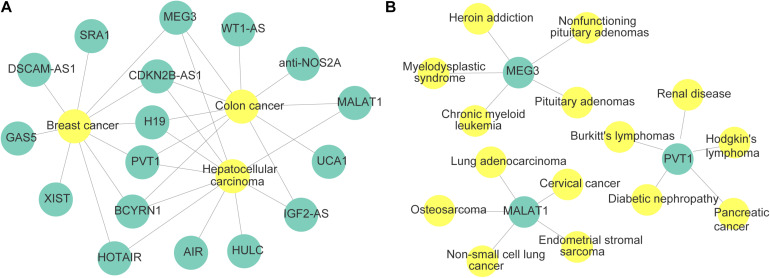
The network of diseases and lncRNAs were made by Cytoscape. **(A)** The network of novel lncRNAs related to colon cancer, hepatocellular carcinoma, and breast cancer. **(B)** The network of novel diseases related to lncRNA MALAT1, PVT1, and MEG3.

Finally, the network of three cases and lncRNAs predicted by MHRWRLDA is shown in Figure 4A; it revealed that MEG3, CDKN2B-AS1, H19, PVT1, BCYRN1, HOTAIR, and all three diseases are related. In addition to exploring lncRNAs related to novel diseases, it is also extremely important to predict diseases related to novel lncRNAs. Therefore, taking lncRNA MALAT1, PVT1, and MEG3 as examples, the predicted top five diseases related to them are listed in Table 5, and their network is shown in Figure 4B. The experimental results proved that MHRWRLDA was useful for predicting the potential lncRNA–disease association.

**TABLE 5:** The predicted top five novel disease correlated with MALAT1, PVT1, and MEG3.

LncRNA	Disease	Rank	Evidence
MALAT1	Endometrial stromal sarcoma	1	Confirmed
	Non-small cell lung cancer	2	Confirmed
	Lung adenocarcinoma	3	Confirmed
	Cervical cancer	4	Confirmed
	Osteosarcoma	5	Confirmed
PVT1	Burkitt’s lymphomas	1	Confirmed
	Hodgkin’s lymphoma	2	Confirmed
	Renal disease	3	Confirmed
	Diabetic nephropathy	4	Confirmed
	Pancreatic cancer	5	Confirmed
MEG3	Pituitary adenomas	1	Confirmed
	Heroin addiction	2	Confirmed
	Nonfunctioning pituitary adenomas	3	Confirmed
	Chronic myeloid leukemia	4	Confirmed
	Myelodysplastic syndrome	5	Confirmed

## Discussion

In recent years, the research on the interaction between biomolecules has been growing. Due to the importance of lncRNA, the research on the associations between lncRNAs and diseases has been paid more and more attention. These associations can be characterized by complex networks, so it is urgent to develop network-based computational algorithms to explore functional associations between lncRNAs and diseases. The algorithm of constructing heterogeneous network and implementing random walk on heterogeneous network is widely used in the field of bioinformatics. However, in previous studies, most of them are single heterogeneous networks with a single information source. Therefore, we consider multiple network embedding by integrating different types of edges. Multiplex and heterogeneous networks are the combination of heterogeneous networks connected by multiple interactions; they integrate the framework of multiple information sources, and each layer is a simplex network with specific types of nodes and edges; when the data set is large, they can produce better results. Multiple heterostructures may provide a richer perspective for the study of the complex relationship between different biological components.

In this study, we extend it to multi-layer heterogeneous networks so as to more effectively predict lncRNA–disease associations. We constitute a multiplex and heterogeneous network by integrating known lncRNA–disease association, lncRNA function similarity, lncRNA Gaussian similarity network, disease semantic similarity network, and disease Gaussian similarity network, and then we generate the final comprehensive predictive scores by the random walk with restart on the multiplex and heterogeneous network, so as to forecast potential lncRNA–disease associations. LOOCV experimental verification results showed that the AUC was 0.68736, which exceeded other algorithms to predict lncRNA–disease association. In novel diseases, the top 10 lncRNAs were verified and predicted by database or literature. In addition, the model can also predict diseases associated with particular lncRNAs.

The network-based approach overcomes the disadvantage of machine learning methods that need to construct negative samples and not only is suitable for predicting lncRNA–disease associations, but also proved to be widely used in exploring disease-related miRNAs, drug repositioning, and prediction of disease–gene associations. Therefore, if the known lncRNA–disease association data are replaced with miRNA-disease association data, MHRWRLDA can be used to predict the potential miRNAs associated with disease; similarly, if it is replaced by drug–disease association data or gene–disease association data, it is possible to make contributions to drug repositioning and the exploration of disease-related genes, respectively. In the future, we will try to apply MHRWRLDA to the above aspects for research.

However, there are some limitations. First, there are only two methods for constructing the similarity network; if the calculation method of the similarity network can be increased, the number of layers in the multi-layer heterogeneous graph can be increased to provide more possibilities for particle migration. Second, the lncRNA–disease association data contain only 117 lncRNAs and 159 diseases, of which there are only 285 pairs of correlations; a small data set may also affect the prediction results. In the future, more association data will be discovered and used to overcome the difficulties caused by the complexity and inconsistency of biological data. In addition, efforts will be made to combine multiple prediction models to achieve more accurate predictions.

## Data Availability Statement

The datasets presented in this study can be found in online repositories. The names of the repository/repositories and accession number(s) can be found below: https://github.com/jibinbin171222/MHRWRLDA.

## Author Contributions

WG conceived, designed, and managed the study. YY and BJ designed the method and wrote the original manuscript. YL and LL revised the original draft. JX wrote the code. BL discussed the proposed method and gave further research. All authors read and approved the final manuscript.

## Conflict of Interest

BJ was employed by Geneis Beijing Co., Ltd. The remaining authors declare that the research was conducted in the absence of any commercial or financial relationships that could be construed as a potential conflict of interest. The handling editor declared a past co-authorship with one of the authors JX.

## Publisher’s Note

All claims expressed in this article are solely those of the authors and do not necessarily represent those of their affiliated organizations, or those of the publisher, the editors and the reviewers. Any product that may be evaluated in this article, or claim that may be made by its manufacturer, is not guaranteed or endorsed by the publisher.
