# Supplementary material for: Predicting LncRNA–Disease Association by a Random Walk With Restart on Multiplex and Heterogeneous Networks
Source: Front Genet. 2021 Aug 19;12:712170. doi: 10.3389/fgene.2021.712170 (PMC8417042; doi:10.3389/fgene.2021.712170)
Supplement: Supplementary file 1 [file Data_Sheet_1.docx]

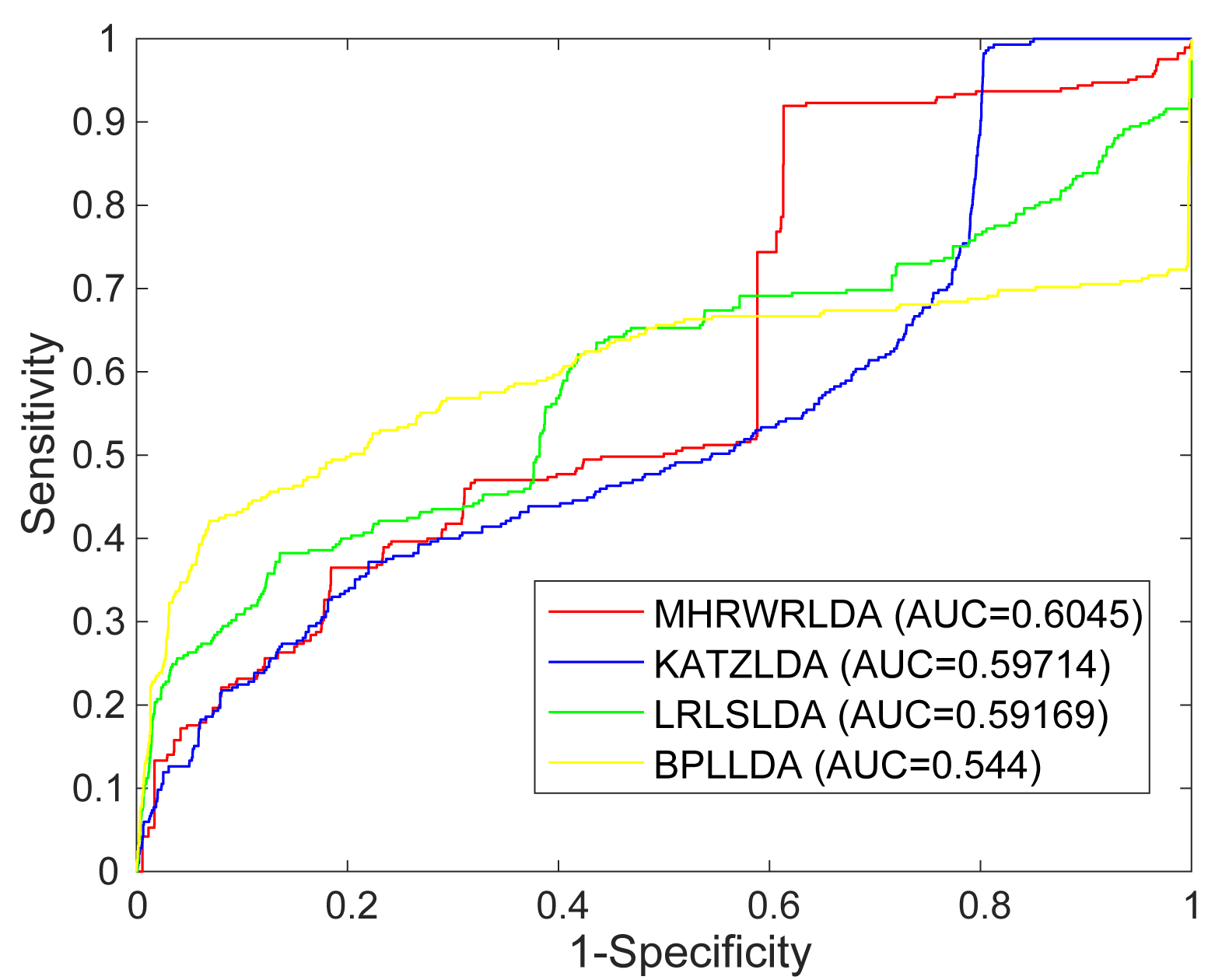


**Figure S1.** **The ROC curves of MHRWRLDA, KATZLDA, BPLLDA and LRLSLDA based on local LOOCV.**


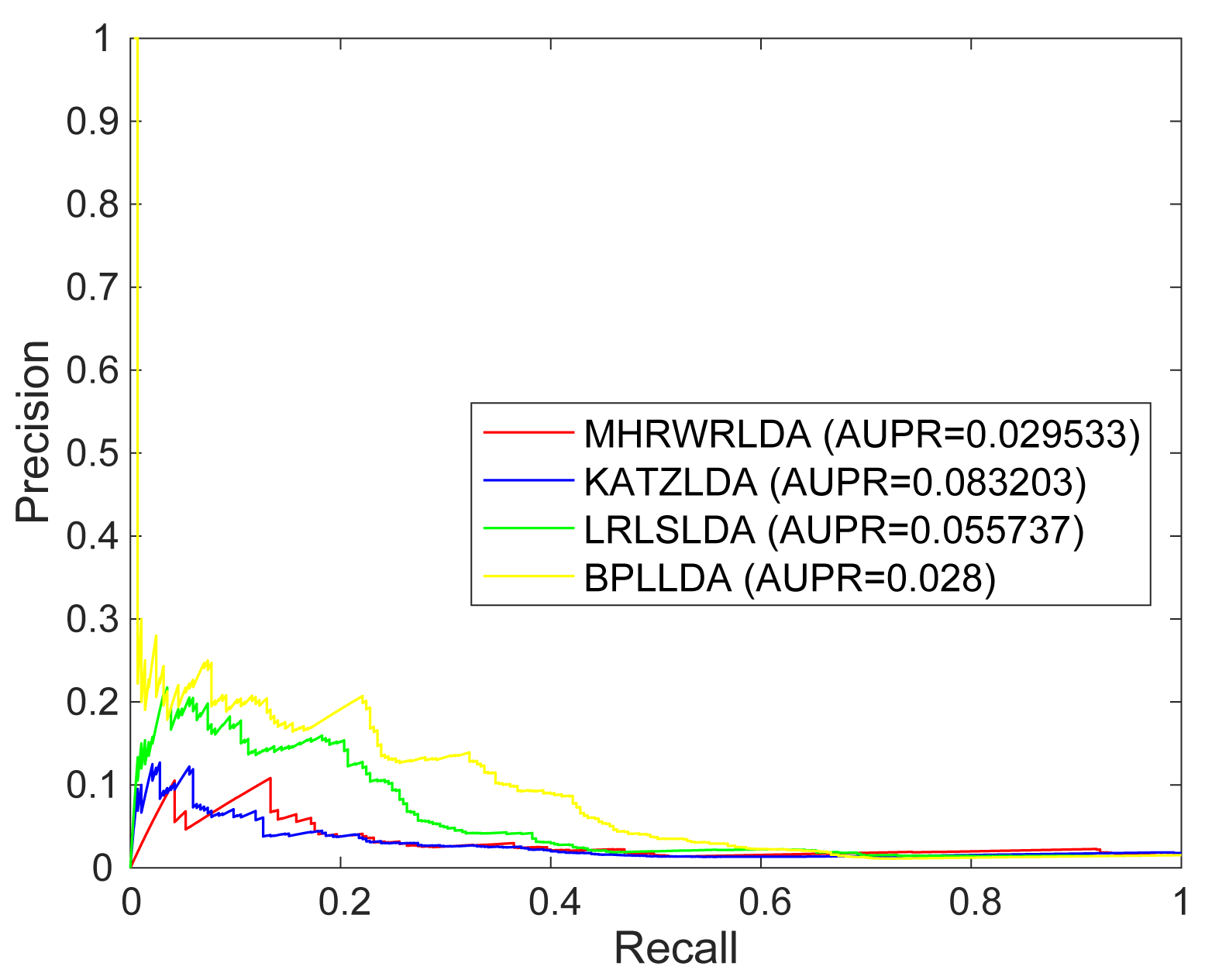


**Figure S2.** **The PR curves of MHRWRLDA, KATZLDA, BPLLDA and LRLSLDA based on local LOOCV.**
